# 2-Ethylhexyl Diphenyl Phosphate Affects Steroidogenesis and Lipidome Profile in Human Adrenal (H295R) Cells

**DOI:** 10.1021/acs.chemrestox.5c00030

**Published:** 2025-04-03

**Authors:** Chander K. Negi, Darshak Gadara, Lola Bajard, Zdeněk Spáčil, Ludek Blaha

**Affiliations:** RECETOX, Faculty of Science, Masaryk University, Kotlarska 2, 61137 Brno, Czech Republic

## Abstract

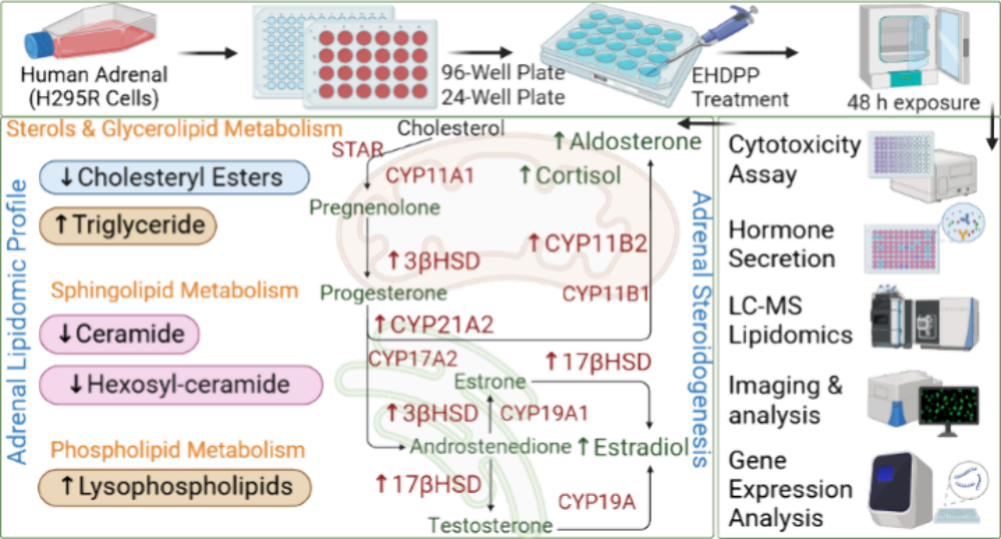

The ever-increasing use of chemicals and the rising incidence of adverse reproductive effects in the modern environment have become an emerging concern. Several studies have shown that environmental contaminants, such as organophosphate flame retardants (OPFRs), negatively impact reproductive health. To evaluate the potential endocrine-related adverse reproductive effects of widely used and priority-listed compound 2-Ethylhexyl diphenyl phosphate (EHDPP), we characterized its effects on adrenal steroidogenesis in human adrenocortical (H295R) cells. The cells were exposed to EHDPP (1 and 5 μM) for 48 h, and the production of hormones, including progesterone, androstenedione, testosterone, estradiol, cortisol, and aldosterone, was measured. In addition, LC-MS/MS-based lipidomics analysis was done to quantify intracellular lipid profiles, and transcriptional assays were performed to examine the expression of genes related to corticosteroidogenesis, lipid metabolism, and mitochondrial dynamics. Our findings indicate that EHDPP disrupts hormone regulation in vitro, as evidenced by increased estradiol, cortisol, and aldosterone secretion. The expression of key corticosteroidogenic genes (CYP11B2, CYP21A1, 3β-HSD2, and 17β-HSD1) was upregulated significantly upon EHDPP exposure. Intracellular lipidomics revealed EHDPP-mediated disruption, including reduced total cholesterol ester, sphingolipids, and increased phospholipids, triglyceride species, and saturated-monounsaturated lipids subspecies. These alterations were accompanied by decreased ACAT2 and SCD1 gene expression. Moreover, a shift in mitochondrial dynamics was indicated by increased MF1 expression and decreased FIS1 expression. These data suggest that EHDPP disrupts adrenal steroidogenesis and lipid homeostasis, emphasizing its potential endocrine-disrupting effects.

## 1 Introduction

Organophosphate flame retardants (OPFRs), including 2-Ethylhexyl diphenyl phosphate (EHDPP), are frequently detected in environmental and biological matrices, such as children’s and adult hand wipes, drinking water, foodstuff, indoor dust, etc.^1−5^ Moreover, these compounds and their metabolites have been identified in human blood, seminal fluid, urine, and even chorionic villi.^6−10^ Their widespread highlights significant human exposure and raises concerns about potential adverse health effects.^11,12^ EHDPP has been reported to affect reproductive function in experimental animals with sex-dependent reproductive alterations observed in zebrafish.^13^ It exhibited androgen receptor antagonistic activity and suppressed androgens-induced intersex development in male Japanese medaka, repressed reproductive behaviors in male fish, and decreased fertility.^14^ More recently, hydroxylated metabolites of EHDPP have been positively associated with alteration in reproductive hormone levels in women of childbearing age,^15^ suggesting possible endocrine-disruption that may compromise reproductive health. However, evidence of the human-relevant reproductive effects of EHDPP remains limited, necessitating further research.

Our previous studies demonstrated that EHDPP induces lipid accumulation in the human liver cell culture^16^ and disrupts lipids homeostasis, including cholesterol esters metabolism in HepG2 3D hepatospheroids.^17^ Additionally, PPARγ-mediated adipogenesis has been observed after EHDPP exposure in mouse 3T3-L1 preadipocytes.^18^ These findings suggest potential lipid-dysregulation effects of EHDPP, which may further contribute to adverse reproductive outcomes, including steroid hormone alteration and impaired fertility. Dysregulation of lipid metabolism is known to significantly influence reproductive function.^19^ Reproductive disorders can disrupt metabolic process, while metabolic dysfunction can similarly impair reproductive function;^20^ however, the underlying molecular mechanisms remain poorly understood. Potential mechanisms include the disruption of the endocrine axis, metabolic signaling, lipid metabolism, and epigenetic modifications.

Lipids play a crucial role in the normal physiological reproductive process. For instance, dysregulation of cholesterol and/or cholesterol esters—a key substrate for steroid hormone biosynthesis—can impair steroidogenesis and potentially affect reproductive behavior.^21^ Given our previous findings on EHDPP-induced lipid dysregulation in human liver cell cultures, we hypothesize that EHDPP may alter the biosynthesis of lipid-derived steroid hormones. To investigate this, we used the human adrenocortical (H295R) cell line, which is capable of producing steroids and their intermediates across all three adrenal cortex zones.^22^ The H295R model is widely recognized for its relevance in reproductive toxicity assessment^23^ and has been validated by the Organisation for Economic Co-operation and Development (OECD) as a standard assay for identifying steroidogenesis disruptors in a regulatory context.^24^ After exposure of EHDPP to the H295R cells, we measured the production of key steroid hormones, such as progestin (progesterone), androgens (testosterone and androstenedione), estrogen (estradiol), and glucocorticoids (cortisol and aldosterone). To elucidate the molecular mechanisms for altered hormone production, we performed transcriptional analysis of genes involved in corticosteroidogenesis, lipid metabolism, and mitochondrial dynamics. Additionally, intracellular lipid levels were quantified using the LC-MS/MS lipidomics platform.

## 2 Materials and Methods

### 2.1 Cell Culture and Exposure

The human adrenocortical carcinoma H295R cell line obtained from the American Type Culture Collection (ATCC) was cultured in phenol red-free Dulbecco’s modified Eagle’s medium plus Ham’s F-12 nutrient mixture DMEM/F12 medium (1:1), containing HEPES buffer and sodium bicarbonate (Sigma) supplemented with 1% insulin-transferrin-selenium-G (ITS-G, Gibco) and 2.5% Nu-Serum (BD Bioscience) at 37 °C in a humidified condition with a 5% CO_2_ atmosphere. The medium was refreshed every third day, and the subculture was performed using 0.25% trypsin–EDTA (Biosera). Cells between passages 5 and 10 after thawing were used for all experiments. 2-Ethylhexyl diphenyl phosphate (EHDPP, >90.0%, CAS 1241-94-7) was purchased from the Tokyo Chemical Industry (TCI, Europe). A working stock solution (100 mM) was prepared by dissolving it in dimethyl sulfoxide (DMSO) (Sigma-Aldrich) and stored at −20 °C. The amount of DMSO for exposure studies was maintained at 0.01%.

### 2.2 Cell Viability Assay

Cell viability was assessed using a combination of three indicator dyes: 5-carboxyfluorescein diacetate acetoxymethyl ester (CFDA-AM; Thermo Fisher Scientific), neutral red (Sigma-Aldrich), and resazurin (Thermo Fisher Scientific) to capture different mechanisms underlying the cytotoxicity. Briefly, 4 × 10^4^ cells/well grown for 24 h in 96-well plates were exposed to different concentrations of EHDPP for 48 h. After the incubation, cells were rinsed twice with PBS and incubated in the dark for 45 min with the resazurin (4% w/v) and CFDA-AM (4 μM) solution prepared in the serum-free cell culture medium. The fluorescence was measured at the ex/em wavelengths 530/595 and 493/541 nm, respectively, using the BioTek Synergy 5. After removal of the resazurin and CFDA-AM, the cell culture was rinsed twice with PBS and incubated for 1 h with the neutral red solution (0.005% w/v) prepared in the serum-free cell culture medium. After incubation, the accumulated neutral red dye was extracted from cells by lysis solution (1% acetic acid and 50% ethanol). The absorbance was measured at wavelengths of 540 nm (absorption maximum) and 690 nm (background) by using the Synergy MX Microplate Reader (BioTek).

### 2.3 Steroid Hormone Measurement

For the steroid assay, 2 × 10^5^ cells/well were seeded in 24-well plates. After the 24 h adherence period, the cells were incubated with EHDPP under the conditions described above for 48 h. After exposure, culture media was collected and stored at −80 °C until further analysis. On the day of analysis, the frozen media was thawed on ice at room temperature, and hormone levels were measured using ELISA according to the manufacturer’s instructions. Progesterone (K00225, Dialab, Germany), androstenedione (K00197, Dialab, Germany), testosterone (K00234, Dialab, Germany), cortisol (K00201, Dialab, Germany), aldosterone (E-EL-0070, Elabscience), and estradiol (KGE014, R&D Biotech, USA) were analyzed.

### 2.4 Intracellular Lipid Droplet Analysis

Intracellular lipid accumulation was analyzed using BODIPY 493/503 (Thermo Fisher Scientific) staining. Briefly, 4 × 10^4^ cells/well were seeded in 96-well plates. After 24 h of adherence, the cells were treated with different concentrations of EHDPP for 48 h. Then, the cells were washed two times with PBS and fixed with 4% paraformaldehyde at room temperature for 10 min, washed twice with PBS, and stained for 30 min with 1.25 μg/mL BODIPY 493/503 and 1.25 μg/mL Hoechst solution prepared in PBS in the dark at 37 °C. About nine images per triplicate well were acquired using BioTek Cytation 5 at 20× magnification. The acquired images were later analyzed using the BioTek Cytation 5-inbuilt automated image analysis tool. The total area stained by BODIPY 493/503 was quantified to measure lipid accumulation. This value was then normalized to the number of nuclei identified by Hoechst staining to assess lipid accumulation per cell.

### 2.5 In-Plate Lipidomics Analysis

For lipidomics analysis, 4 × 10^4^ cells/well were seeded in 96-well plates and allowed to adhere for 24 h. The cells were then exposed to subcytotoxic concentrations of EHDPP for 48 h. Following treatment, the culture media was aspirated carefully, and the cells were washed twice with PBS to remove any residual compounds. Lipids were then extracted following the protocol described previously.^17^ Briefly, 120 μL of isopropanol (Biosolve) containing a mixture of 16 internal lipid standards (SPLASH Lipidomics; cat. #330707) and internal sphingolipid standards (Cer/Sph Mixture I, Avanti Polar Lipids, Alabaster, USA cat. #LM6002) was added to each well to extract the lipids. A silicone cap-mat lined with polytetrafluoroethylene (PTFE) was glued to each 96-well plate and then subjected to sonication (15 min), vortexing (5 min), and centrifugation (10 min). Lipid extract (70 μL) was transferred into a new 96-well sample collection plate (Waters), and the plate was placed directly into the autosampler of the 1290 Infinity II UHPLC (Agilent) system coupled to the 6495 Triple Quadrupole mass spectrometer (Agilent). Lipid extracts (2 μL) were injected onto a reversed-phase microbore column (CSH, 1 mm × 100 mm, 1.7 μm, Waters) and separated at a flow rate of 100 μL/min over 15 min. To ensure analytical robustness, prevent carryover, and maintain column and ion source integrity, solvent blanks were injected after every four sample runs. Pooled quality control (QC) samples, prepared by combining extracts from all study samples, were analyzed after every eight injections to monitor the analytical performance and reproducibility. All samples were analyzed in a randomized sequence to minimize the systematic variation. Chromatographic separation was performed by using a linear gradient elution. Mobile phase A consisted of 10 mM ammonium formate (Sigma-Aldrich) in acetonitrile:water (60:40), while mobile phase B contained 10 mM ammonium formate in isopropanol:acetonitrile (90:10, Honeywell). The gradient elution was programmed as follows: 15% B at 0 min, increasing to 30% B at 1.86 min, 48% B at 2.32 min, 82% B at 9.5 min, followed by 99% B from 12.5 to 13.5 min, with column re-equilibration until 15 min. Electrospray ionization (ESI) was performed in positive mode with the following parameters: gas temperature, 200 °C; gas flow, 14 L/min; nebulizer pressure, 45 psi; sheath gas temperature, 400 °C; sheath gas flow, 8 L/min; capillary voltage, 4 kV; nozzle voltage, 500 V; and unit resolution for Q1 and Q3. The mass spectrometer operated in dynamic selected reaction monitoring (SRM) mode with a 2 min retention time window per transition.

For lipidomic data processing, raw files were analyzed by using MassHunter Quantitative Analysis software (B.07.00, Agilent Technologies). A predefined list of lipid species was curated and quantified using selected reaction monitoring (SRM) transitions reported in the literature and validated with quality control (QC) samples.^25−27^ The list of SRM is presented in Supporting Information, Table S5. Collision energy optimization was performed through the direct infusion of 16 class-specific internal standards. Lipid species were annotated according to LIPID MAPS consortium guidelines with total carbon number and degree of unsaturation indicated (e.g., PC 34:2). In the case of diglycerides (DG) and triglycerides (TG), individual acyl chain compositions were specified (e.g., TG 48:2/18:1). Identification of lipid species was based on characteristic retention behaviors of carbon chain length and degree of unsaturation.^28^ Absolute lipid quantification was performed relative to internal standards (Table S1). Only lipid species with a signal-to-noise ratio greater than three and a coefficient of variation (%CV) below 30% were considered for further biological interpretation.

### 2.6 Gene Expression Analysis

Total RNA was extracted using the Quick-RNA Microprep Kit (Zymo Research) according to the manufacturer’s protocol, and quality was verified spectrophotometrically at 260 nm by using a nanodrop spectrophotometer (Thermo Fisher Scientific, USA). RNA was then reverse transcribed using a SensiFAST cDNA synthesis kit (Bioline). The resulting cDNA was amplified by RT-qPCR using a SYBR Green PCR master mix (ThermoFisher Scientific, Waltham, MA) in Roche light cycler 480. The RT-qPCR conditions were set at 95 °C for 15 min and 40 cycles of 10 s at 95 °C, 20 s at 60 °C, and 32 s at 72 °C. Melting curve analyses were performed immediately following the final PCR cycle to differentiate between the desired amplicons and any primer dimers or DNA contaminants. Expression of genes encoding for cytochrome P450 family (CYP11A, CYP11B2, CYP17A1, CYP19A1, CYP21A2), hydroxysteroid dehydrogenases (3βHSD2, 17βHSD1, 17βHSD4), steroidogenic acute regulatory protein (STAR), and 3-hydroxy-3-methylglutaryl coenzyme A reductase (HMGCR), lipid metabolism-related genes (HMGCR, ACAT1, ACAT2), and mitochondrial dynamics-related genes DRP1, MFN1, MFN2, OPA1, and FIS1 were analyzed. Primer sequences have already been published,^29^ also listed in Table 1. The expression levels of target genes were normalized to the geometric mean of two reference genes: 18S rRNA and β-actin mRNA levels. The relative mRNA levels of genes were quantified according to Livak and Schmittgen’s method.^30^

**Table 1:** Primer Pairs Used in Real-Time Quantitative Polymerase Chain Reactions

gene	forward primer	reverse primer
18S rRNA	CGTCTGCCCTATCAACTTTCG	TGCCTTCCTTGGATGTGGTAG
β-actin	CACTCTTCCAGCCTTCCTTCC	AGGTCTTTGCGGATGTCCAC
CYP11A	GAGATGGCACGCAACCTGAAG	CTTAGTGTCTCCTTGATGCTGGC
CYP11B2	TCCAGGTGTGTTCAGTAGTTCC	GAAGCCATCTCTGAGGTCTGTG
CYP17	AGCCGCACACCAACTATCAG	TCACCGATGCTGGAGTCAAC
CYP19	AGGTGCTATTGGTCATCTGCTC	TGGTGGAATCGGGTCTTTATGG
CYP21	CGTGGTGCTGACCCGACTG	GGCTGCATCTTGAGGATGACAC
3βHSD2	TGCCAGTCTTCATCTACACCAG	TTCCAGAGGCTCTTCTTCGTG
17βHSD1	CTCCCTCTGACCAGCAACC	TGTGTCTCCCACGCAATCTC
17βHSD4	TGCGGGATCACGGATGACTC	GCCACCATTCTCCTCACAACTC
StAR	GTCCCACCCTGCCTCTGAAG	CATACTCTAAACACGAACCCCACC
HMGR	TGCTTGCCGAGCCTAATGAAAG	AGAGCGTTCGTGGGTCCAT
ACAT2	CTTTAGCACGGATAGTTTCCTGG	GCTGCAAAGGCTTCATTGATTTC
ACAT1	ATGCCAGTACACTGAATGATGG	GATGCAGCATATACAGGAGCAA
SCD1	CCTTATGACAAGAACATTAGCCCC	GGTGAAGTTGATGTGCCAGC

### 2.7 Statistical Analysis

All data are expressed as the mean ± the standard error of the mean (SEM) from three independent experiments. Lipidomic data were processed using MassHunter Quantitative Analysis software (B.07.00, Agilent Technologies). Absolute lipid quantification was performed relative to internal standards, and only lipid species with a signal-to-noise ratio greater than 3 and a coefficient of variation (%CV) less than 30% were included in the analysis. Statistical analysis and data visualization, including principal component analysis (PCA), boxplots, and heatmaps, were conducted using GraphPad Prism version 9 (GraphPad Software, La Jolla, California, USA, www.graphpad.com). Statistical comparisons between experimental groups were performed using one-way analysis of variance (ANOVA) followed by Dunnett’s multiple comparisons test. A *p*-value <0.05 was considered statistically significant.

## 3 Results

### 3.1 EHDPP Induces Adrenal Cell Death at High Micromolar Concentration

**Figure 1:**
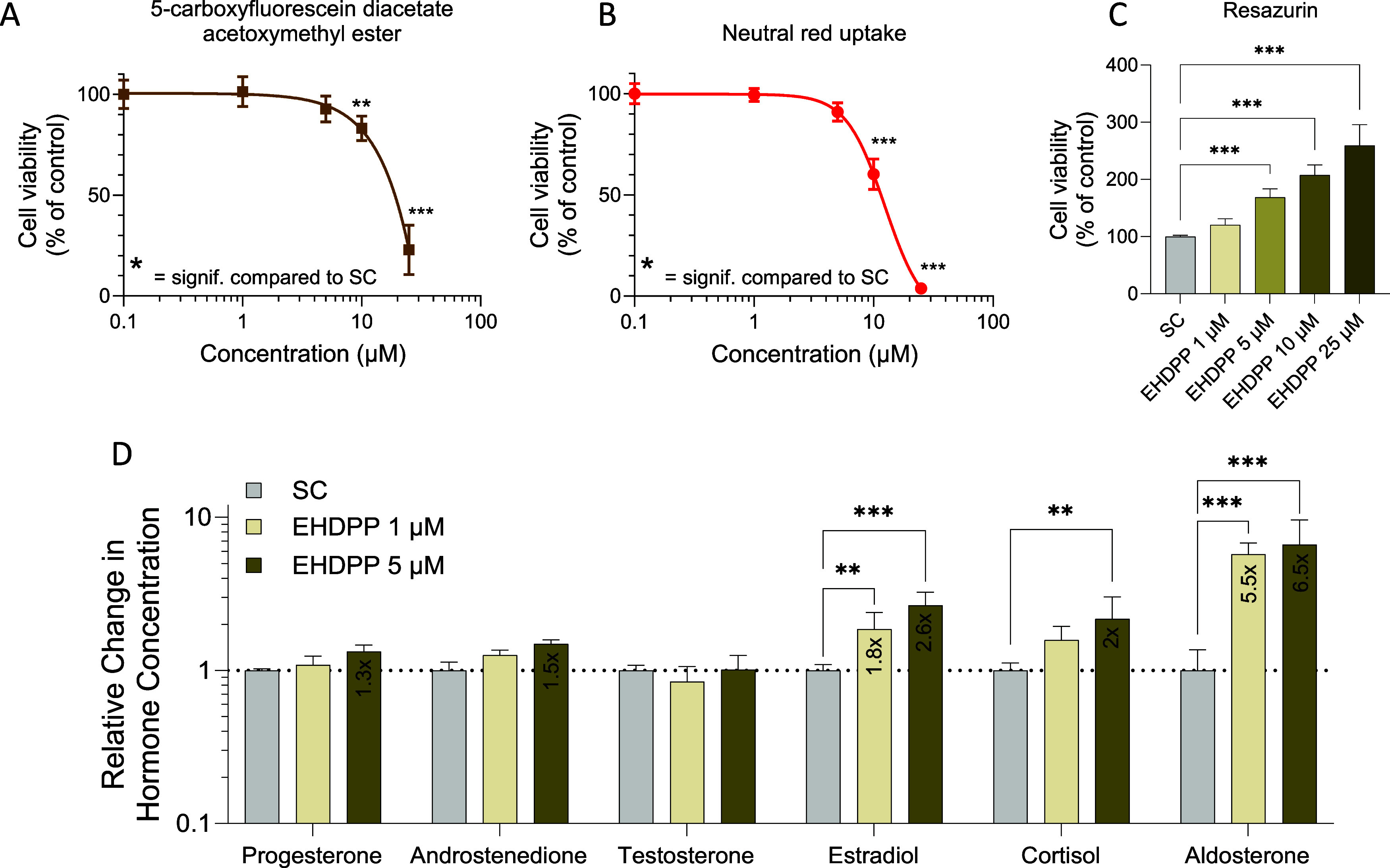
(A) Effects of EHDPP on cell viability assessed by three indicator dyes: (A) 5-carboxyfluorescein diacetate acetoxymethyl ester (CFDA-AM), (B) neutral red, and (C) resazurin and (D) hormone secretion in the human adrenal (H295R) cells after exposure to EHDPP for 48 h. Values are expressed as mean ± SEM of three independent experiments (*n* = 3). The asterisks indicate statistically significant differences from SC, *p* < 0.01 (**) and ***p < 0.001.

EHDPP demonstrated cytotoxic effects at higher micromolar concentrations in human adrenalin (H295R) cells. While no effects were observed on cytoplasmic esterase activity (as measured by CFDA-AM) for up to 10 μM, higher 25 μM concentration caused significant inhibition, leading to a marked decrease in fluorescence intensity indicative of potential cytotoxicity or metabolic stress (Figure 1A). No cytotoxicity was observed in the neutral red assay up to 5 μM, and a reduction in the lysosomal activity emerged at 25 μM (Figure 1B). In contrast, the resazurin assay, which assesses mitochondrial activity, revealed a concentration-dependent increase starting at 5 μM (Figure 1C). Based on the results, the 1 and 5 μM concentrations, which represent the subcytotoxic range, were used in further experiments.

### 3.2 EHDPP Affects Adrenal Steroidogenesis

To assess the effects of EHDPP on steroidogenesis, we measured adrenal-secreted hormones such as progestin (progesterone), androgens (testosterone and androstenedione), estrogen (estradiol), and glucocorticoids (cortisol and aldosterone). Exposure to EHDPP for 48 h significantly increased the secretion of estradiol, cortisol, and aldosterone (Figure 1D), while no significant change in the secretion of progesterone, androstenedione, and testosterone was observed.

### 3.3 EHDPP-Induced Accumulation of Neutral Lipid Droplets

**Figure 2:**
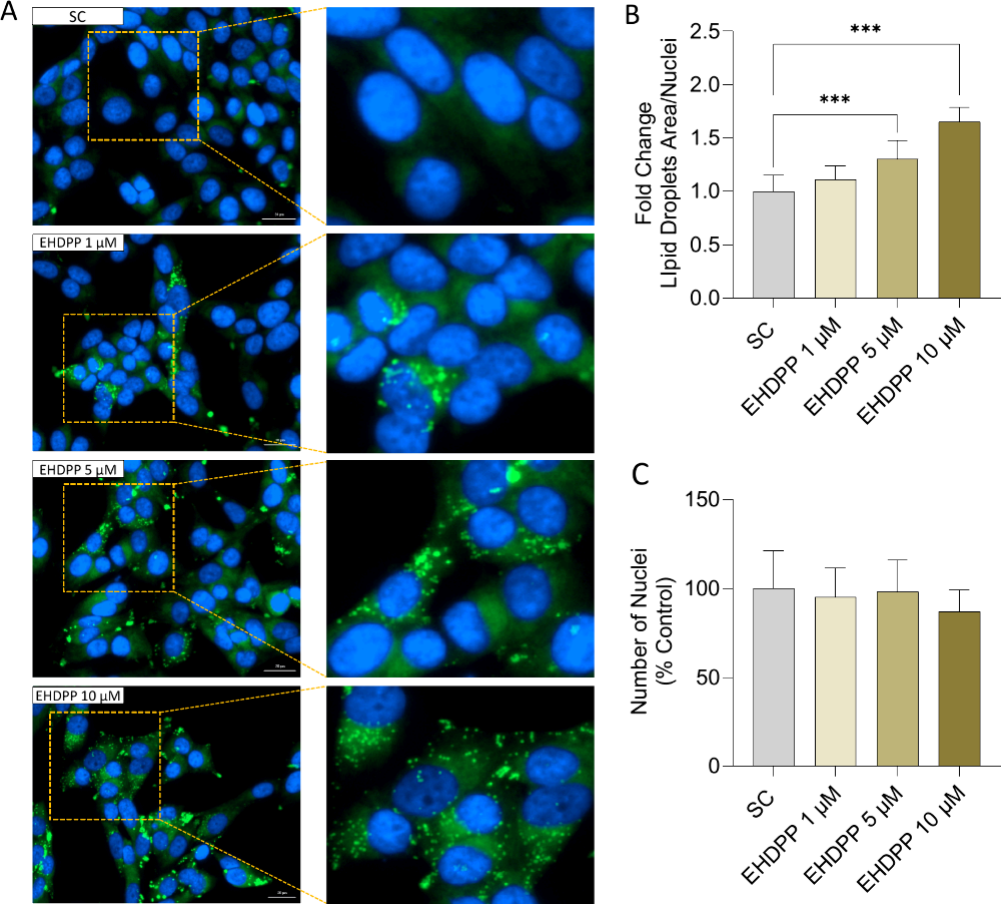
Effects of EHDPP on neutral lipid accumulation in H295R cells after 48 h of exposure. (A) Representative photomicrographs of BODIPY 493/503 staining (B) Quantitative analysis of (A) lipid droplets and (C) nuclei, blue. Values are expressed as mean ± SEM of three independent experiments (*n* = 3). The asterisks indicate statistically significant differences from SC, *p* < 0.01 (**) and ***p < 0.001.

The accumulation of lipid droplets was assessed using BODIPY 493/503 and a Hoechst double staining process. The high-content imaging analysis indicated that exposure to EHDPP for 48 h resulted in a significant increase in lipid accumulation in a dose-dependent manner in H295R cells (Figure 2). Notably, the number of nuclei remained consistent across all treatment groups, indicating that the observed increases in lipid accumulation were not due to differences in cell proliferation but instead reflected an increase in the neutral lipid content within the cells.

### 3.4 EHDPP Alters the Lipid Profile of Human Adrenal Cells

**Figure 3:**
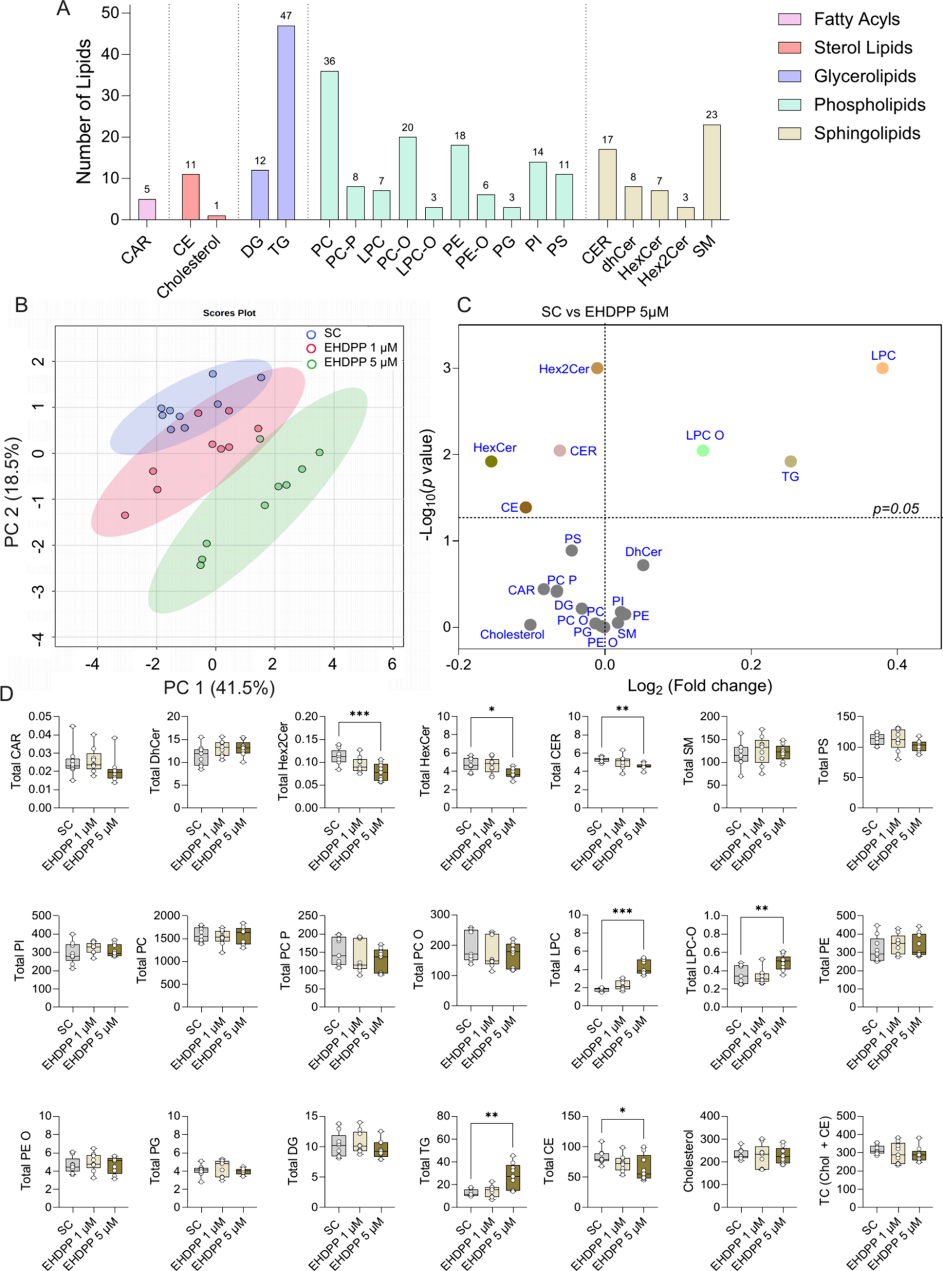
Effects of EHDPP on the intracellular lipid profile of H295R cells. (A) Total number of lipids quantified in each lipid subclass. (B) PCA scores plot of LC-MS/MS-based lipid profiles from SC- or EHDPP (1 and 5 μM)-treated H295R cells. (C) Volcano plot comparing SC (0.01% DMSO) and EHDPP (5 μM) treatments (−log_10_ of the *p*-value is plotted against the log_2_ fold change). (D) Lipid levels (ng/well) presented as box plots, showing measurements from three independent experiments, each performed in triplicate (*n* = 9). Asterisks indicate statistically significant differences from SC: *p < 0.05, **p < 0.01.

**Figure 4:**
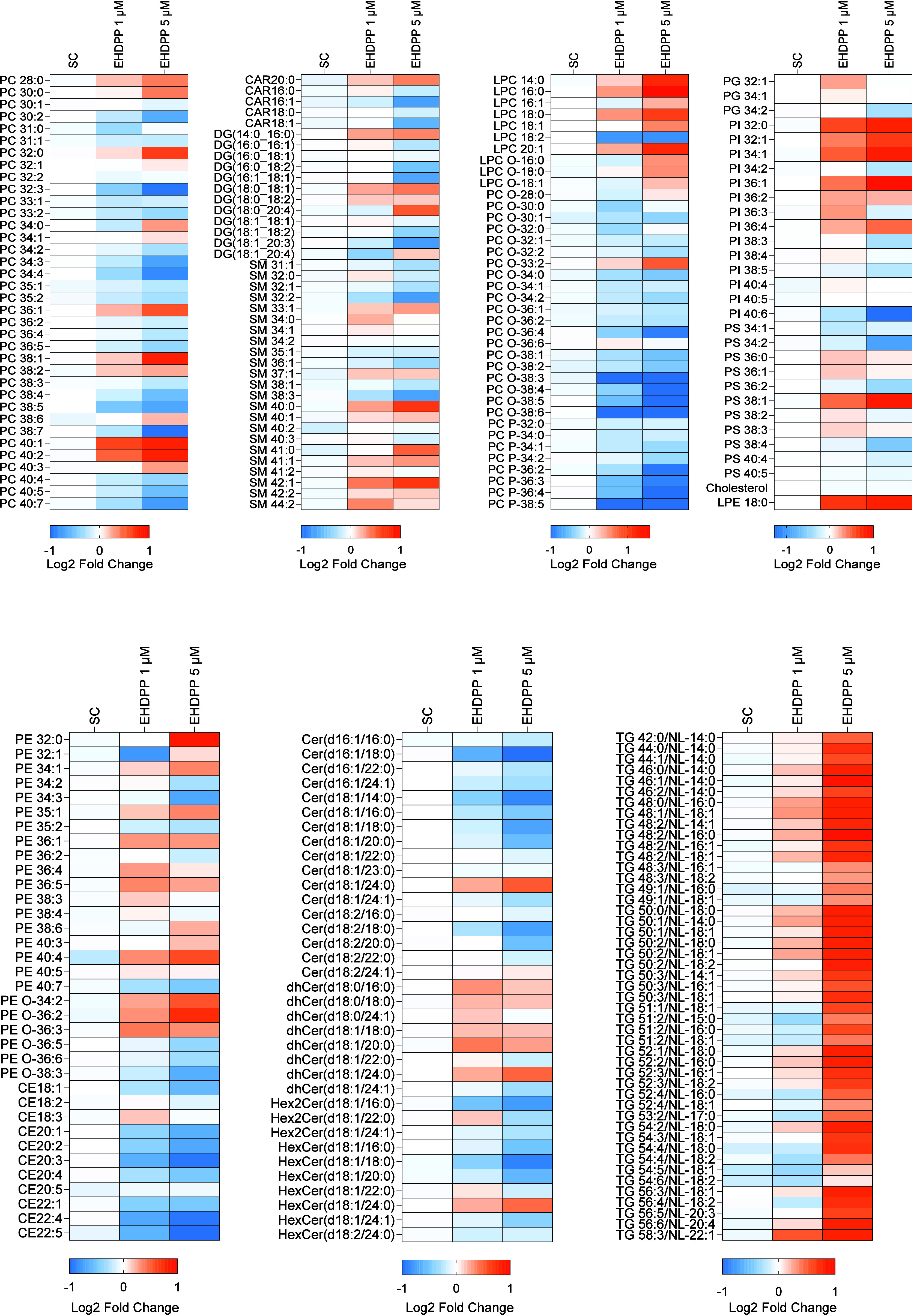
. Heatmap of log_2_ fold changes in lipidomics profiles of H295R cells treated with EHDPP (1 and 5 μM) for 48 h. Lipid abundances were quantified using LC-MS/MS and normalized to internal standards. Log_2_ fold changes were calculated relative to the solvent control group. Each row represents a distinct lipid species, categorized by lipid class. Color intensity indicates the magnitude of change, with orange representing upregulated lipids and blue representing downregulated lipids. Data represent the mean of 9 replicates per condition.

**Figure 5:**
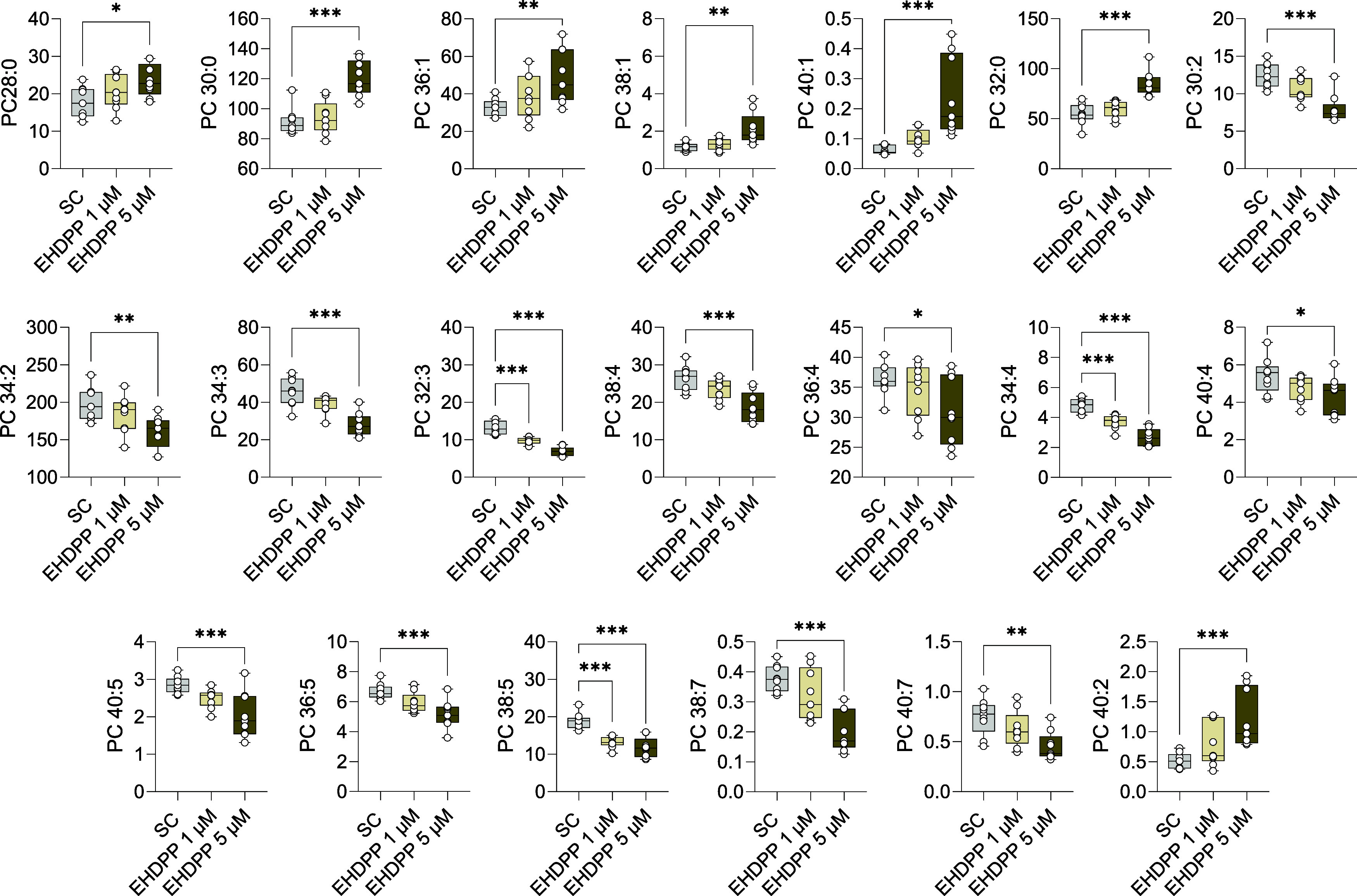
Effects of EHDPP on lipid subclass phosphatidylcholines (PC). Box plots showing the absolute quantification (ng/well) of PC species from three independent experiments, each performed in triplicate, (*n* = 9). The asterisks indicate statistically significant differences from the SC, *p* < 0.05 (*), *p* < 0.01 (**), *p* < 0.001 (**).

**Figure 6:**
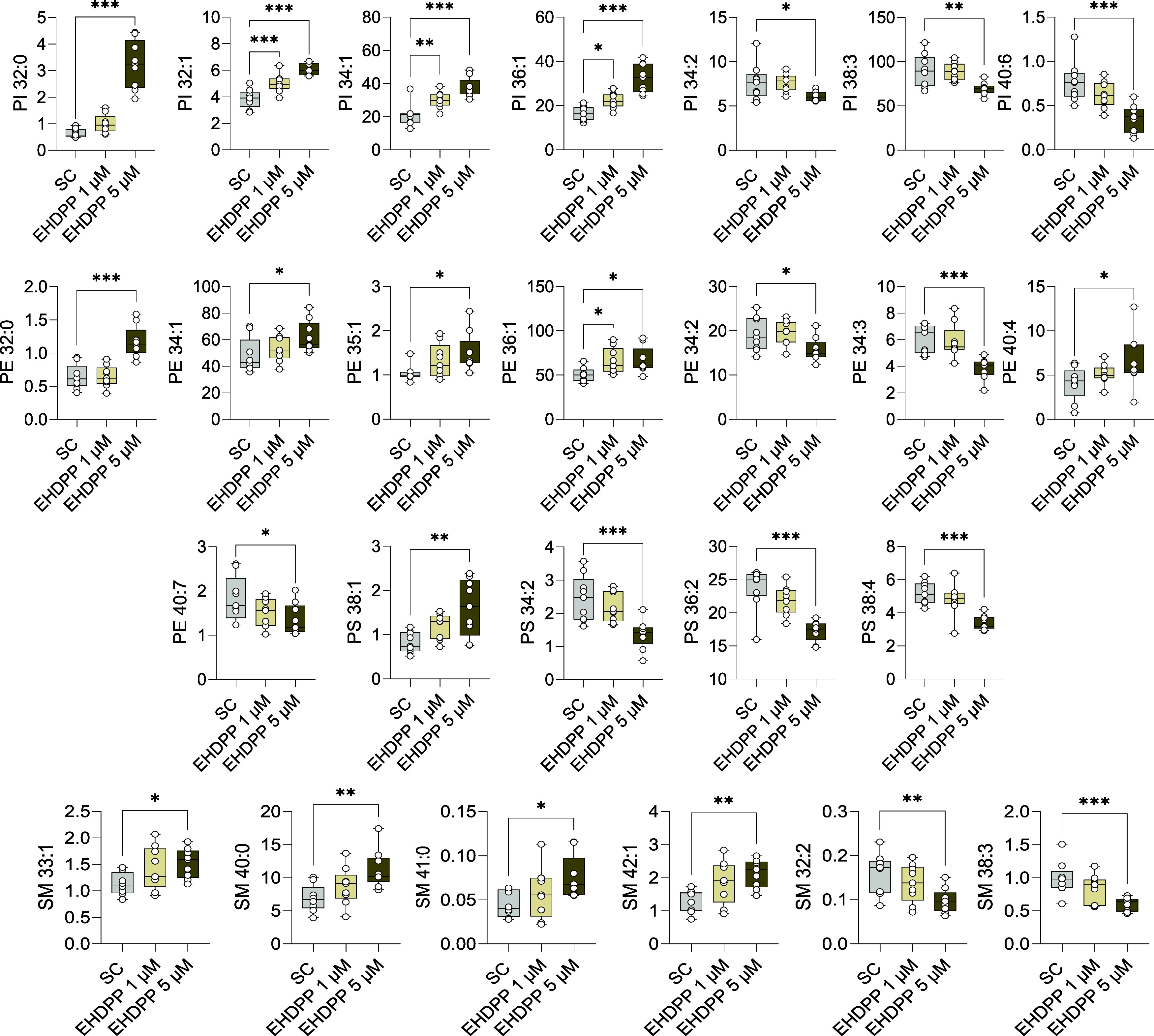
Effects of EHDPP on lipid subclass phosphatidylinositol (PI), phosphatidylethanolamine (PE), phosphatidylserine (PS), and sphingomyelins (SM). Box plots showing the absolute quantification (ng/well) of PE, PS, and SM species from three independent experiments, each performed in triplicate, (*n* = 9). The asterisks indicate statistically significant differences from the SC, *p* < 0.05 (*), *p* < 0.01 (**), *p* < 0.001 (**).

We quantified the intracellular lipid profile of H295R cells after EHDPP exposures using the LC-MS/MS lipidomics platform. As shown in the principal component analysis (PCA) (Figure 3B), EHDPP widely affected the intracellular lipid composition of H295R cells. Detailed analysis showed that it significantly increased the total glycerophospholipid class (lysophosphatidylcholine; LPC, LPC O), whereas total sphingolipids (ceramides: HexCer, Hex2Cer, and CER) were significantly downregulated. EHDPP treatment significantly increased triglycerides (TG) and decreased cholesterol esters (CE), while no significant difference in diglycerides (DG) and free cholesterol FC was noted (Figure 3D). The overall changes in lipidome across all quantified species are visualized in the heatmap (Figure 4), highlighting broad alterations in lipid profiles following EHDPP exposure. Further quantitative analysis of individual lipid species within each class revealed a significant decreasing trend in lipids containing unsaturated acyl chains. For instance, decrease in sphingomyelin species (SM32:2 and SM38:3) while increase in saturated-monounsaturated lipids (SM33:1, SM40:0, SM41:0, SM42:1) was observed (Figure 6). Moreover, similar trends in the phospholipid species were observed as EHDPP significantly decreased PC30:2, PC32:3, PC30:2, PC34:3, PC34:4, PC38:5, PC38:7, and PC40:7, while saturated phospholipids including PC32:0, PC36:1, PC38:1, and PC40:, were decreased (Figure 5). Total PI showed no significant changes; however, saturated PI species (PI32:0, PI32:1, PI34:1, PI36:1) were enhanced at the same time, and unsaturated species, including PI40:6, were decreased significantly (Figure 6). Similarly, PS34:2, PS36:2, and PS38:4 were decreased, while saturated PS38:1 was significantly increased (Figure 6). PE32:0, PE34:1, PE35:1, and PE36:1 were increased, while PE34:2, PE34:3, and PE40:7 were decreased. The observed patterns in the lipidome profile indicate several cellular and metabolic effects following EHDPP exposure such as defects in membrane integrity, altered lipid signaling, and possible adaptive responses to oxidative stress, which may collectively impact hormone synthesis pathways in H295R adrenal cells.

### 3.5 EHDPP Affects the Expression of Corticosteroidogenic, Lipid Metabolism, and Mitochondrial Dynamics-Related Genes

**Figure 7:**
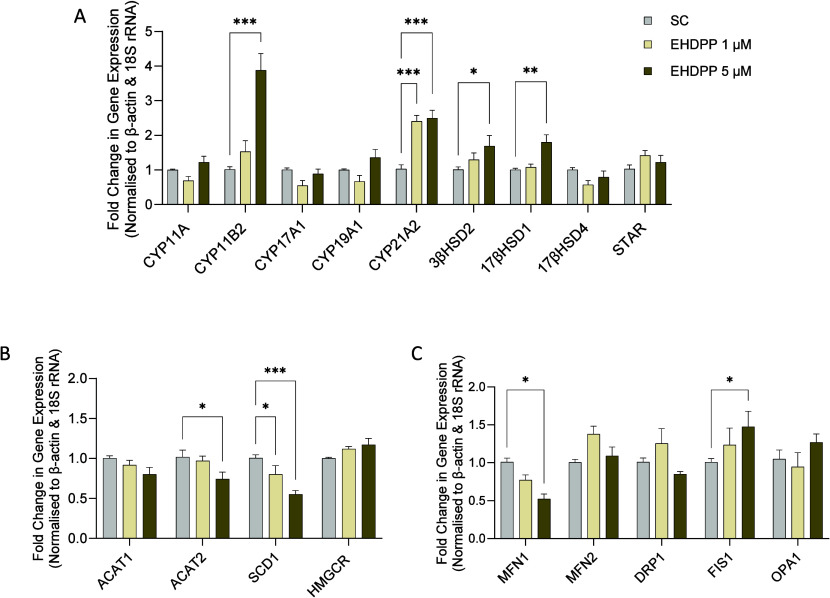
Effects of EHDPP on (A) corticosteroidogenic (B) lipid metabolism-related, and (C) mitochondrial dynamics-related genes expression in H295R cells after 48 h exposure. Values are expressed as mean ± SEM of three independent experiments (*n* = 3). The asterisks indicate statistically significant differences from SC, *p* < 0.05 (*), *p* < 0.01 (**), *p* < 0.001 (**).

To investigate the potential mechanisms for EHDPP-mediated adrenal steroidogenesis, we measured the corticosteroidogenic genes, including CYP11A, CYP11B2, CYP17A1, CYP19A1, CYP21A2, 3βHSD2, 17βHSD1, 17βHSD4, STAR, and lipid metabolism-related genes (ACAT1, ACAT2, SCD1). As shown in Figure 7A,B, EHDPP significantly increased the expression of cytochrome P450-encoding mRNA (CYP11B2, CYP21A1) and 3β-HSD2 and 17β-HSD1 and decreased the expression of ACAT2 (an enzyme in the cholesterol synthesis pathway) and SCD1 which is the rate-limiting enzyme catalyzing the biosynthesis of monounsaturated fatty acids, while no significant change in CYP11A, CYP17A1, CYP19A1, 17β-HSD4, STAR, and HMGCR was observed. In the analysis of mitochondrial dynamics, we observed a significant decrease in the expression of the MFN1 (Mitofusin 1) gene, which is crucial for mitochondrial fusion, while the expression of the FIS1 (Fission 1) gene was notably increased. FIS1 is essential for mitochondrial fission, suggesting a shift toward mitochondrial fragmentation after EHDPP exposure. Interestingly, no significant changes were detected in the expression of DRP1 (Dynamin-related protein 1), MFN2 (Mitofusin 2), or OPA1 (Optic atrophy 1) (Figure 7C), which are also key regulators of mitochondrial fusion and fission, suggesting potential imbalance between fusion and fission processes.

## 4 Discussion

**Figure 8:**
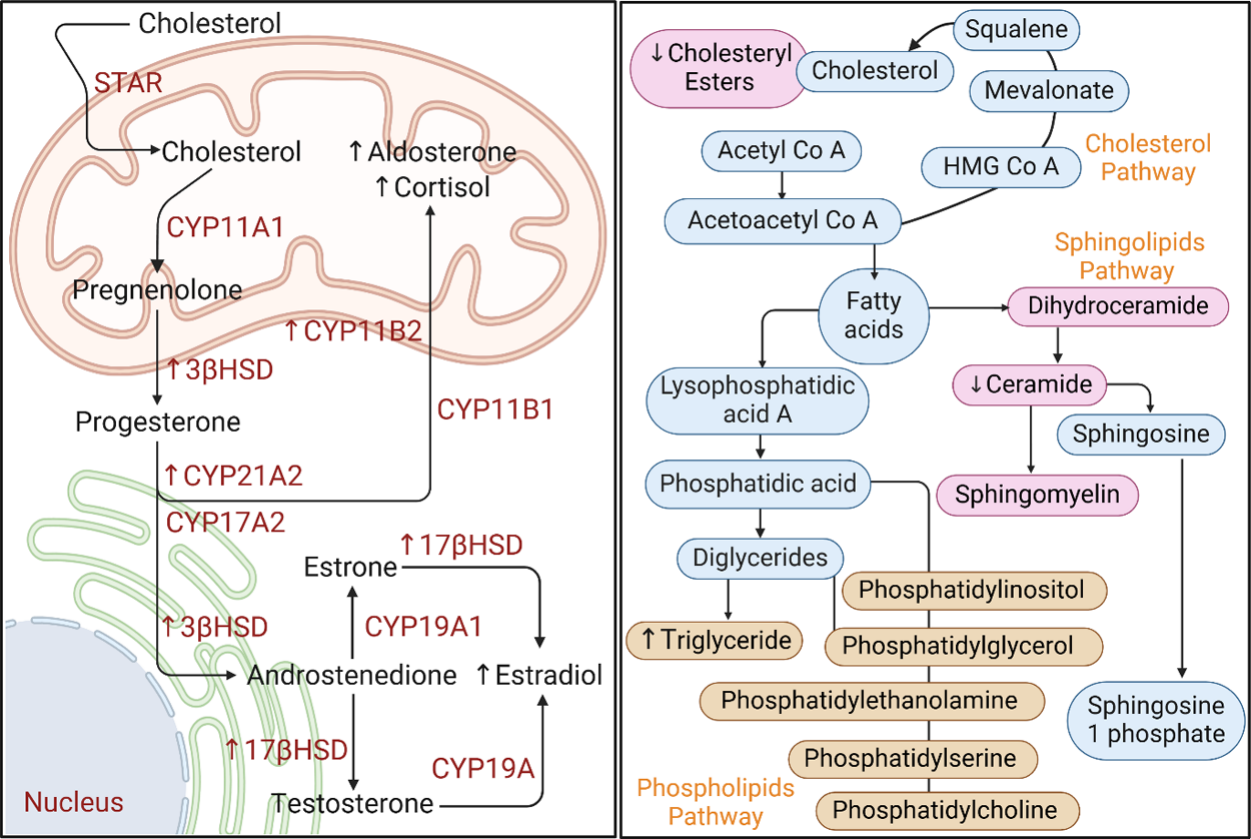
Summary of proposed mechanisms for EHDPP-induced adrenal steroidogenesis and lipid metabolism dysregulation.

EHDPP is one of the most widely used OPFRs, and it has been ubiquitously detected in several human and environmental matrices. Previous studies have reported the antiandrogenic effect of EHDPP in vitro in MDAkb2 cell culture^31^ and its hydroxylated metabolites, which could contribute to the intersex incidence, repression of reproductive behavior, and decreased fertility observed in the study with male Japanese Medaka (*Oryzias latipes*).^14^ Other studies report endocrine-disrupting effects in the murine cell culture.^32^ Considering this, we employed a human-relevant model, the steroidogenic adrenal gland-derived H295R cells, to assess the hormonal dysregulating effects of EHDPP, and the results collected support a proposed mechanism depicted in Figure 8.

In this study, exposure to EHDPP for 48 h significantly increased key steroid hormone secretion in human adrenal (H295R) cells. Specifically, EHDPP exposure led to enhanced secretion of cortisol, aldosterone, and estradiol, suggesting that EHDPP may interfere with the normal steroidogenic processes in these cells. However, despite these alterations in glucocorticoids and mineralocorticoids, EHDPP did not significantly affect the secretion of progesterone, androstenedione, or testosterone, indicating that its effects may be specific to certain steroid pathways. Notably, the elevated secretion of estradiol following EHDPP exposure suggests potential estrogenic activity, which is consistent with findings from previous studies demonstrating that EHDPP mediates activation of the estrogen receptor (ER) and subsequent downstream signaling.^33^ These results suggest that EHDPP may function as an endocrine disruptor, influencing estrogen-related processes. Estradiol, a key estrogen involved in reproductive and metabolic processes, is synthesized through the conversion of androgens into estrogens by aromatase. The EHDPP-mediated increase in estradiol suggests that this compound may interfere with aromatase activity, thereby enhancing the conversion of testosterone to estradiol. The EHDPP-mediated increase in estradiol suggests that this compound may interfere with aromatase activity, thereby enhancing the conversion of testosterone to estradiol. Supporting this, EHDPP has been shown to induce vitellogenin 2 (vtg2) expression, which is a precursor of egg yolk in oviparous animals and indicates estrogenic activity as seen in medaka fish, where plasma 17β-estradiol levels were elevated.^14^ Another study in zebrafish also reported an increase in plasma 17β-estradiol levels, with a more pronounced effect in males, indicating a possible sex-dependent action of EHDPP.^13^ Notably, aromatase (CYP19) plays a crucial role in this process, where exposure to estrogens can induce aromatase expression, promoting the conversion of testosterone to estradiol. Nevertheless, in the present study, we did not observe significant changes in the expression of CYP19 mRNA in H295R cells. It would be valuable to investigate the potential effects of EHDPP on CYP19 expression and activity in more detail in various models.

Moreover, EHDPP exposure also enhanced the glucocorticoid/mineralocorticoid biosynthesis pathway, as evidenced by significantly upregulated mRNA expression of key enzymes of cortisol and aldosterone synthesis, including 3βHSD2, CYP21A2, and CYP11B2. This upregulation was potentially associated with increased synthesis of aldosterone and cortisol. The increased cortisol and aldosterone production in H295R cells further indicates EHDPP influencing the activity of glucocorticoid or mineralocorticoid receptors. Although EHDPP did not alter glucocorticoid receptor in other cell models,^34^ our findings align with previous studies showing that environmental contaminants, including organophosphate esters, can interfere with adrenal steroidogenesis and enhance glucocorticoid and mineralocorticoid biosynthesis.^35^ The upregulation of 3β-HSD2, CYP21A2, and CYP11B2 suggests a disruption in steroidogenic pathways, as these enzymes regulate key steps in cortisol and aldosterone biosynthesis.^36^ 3β-HSD2 catalyzes the conversion of pregnenolone to progesterone, a necessary precursor for corticosteroid production, while CYP21A2 facilitates the 21-hydroxylation of steroid intermediates, a critical step in cortisol and aldosterone synthesis. Additionally, CYP11B2, the aldosterone synthase enzyme, was significantly upregulated, suggesting increased mineralocorticoid production.

Despite the alterations in cortisol, aldosterone, and estradiol, EHDPP does not significantly affect the secretion of progesterone, androstenedione, or testosterone in the present study. In contrast, previous study reports that EHDPP 10 and 20 μM (48 h) significantly enhanced the production of progesterone and promoted human chorionic gonadotropin (hCG) production, respectively, in the human placental choriocarcinoma cell line (JEG-3).^37^ Progesterone is a precursor in the steroidogenesis pathway and is vital to reproductive health. Similarly, androstenedione is a precursor to both testosterone and estrogen which are produced in minor quantities in the adrenal cortex (zona reticularis).^38^ The lack of significant changes in these hormones suggests that EHDPP may selectively affect specific pathways in steroidogenesis, possibly by influencing enzymes CYP11B2, CYP21A1 which are involved in cortisol and aldosterone biosynthesis. 3β-HSD2 is the enzyme that catalyzes the conversion of pregnenolone to progesterone and dehydroepiandrosterone (DHEA) to androstenedione, which are critical steps in the production of both glucocorticoids (cortisol and aldosterone) and androgens.^36^ The increased expression of 3β-HSD2 would theoretically lead to higher progesterone production, but the fact that no significant change in progesterone secretion was observed suggests that the progesterone is rapidly metabolized rather than accumulating as a free hormone and converted into cortisol or aldosterone, which were observed to be elevated. Furthermore, elevated transcription may not always correlate with protein levels and/or enzymatic activities, and further studies are needed to confirm this. 17β-HSD1 enzyme is involved in converting androstenedione to testosterone and estrone to estradiol.^39^ However, its increased expression may significantly impact estradiol synthesis due to its strong association with converting estrone to estradiol.^39^ As observed, the increase in estradiol secretion is consistent with the upregulation of 17β-HSD1 expression. Despite this, the lack of significant changes in testosterone levels suggests that other regulatory mechanisms or pathways control testosterone production, potentially limiting the conversion of androstenedione to testosterone while favoring estradiol synthesis. At the same time, no significant change in androgen (testosterone) indicates the potential antiandrogenic activity of EHDPP, which has been demonstrated previously using MDAkb2 cell culture.^31^ The H295R cells in the present study were derived from female adrenal tissue and are thus more prone to synthesizing glucocorticoids (cortisol) and mineralocorticoids (aldosterone) over androgens.^40^ Female adrenals may show distinctive steroidogenic activity that can differ from male adrenals, including male adrenals or testes.

Lipids are an essential cellular component that also regulates endocrine, metabolic, and reproductive functions. It has been shown that EHDPP affects lipid metabolism^17^ and induces the accumulation of neutral lipid droplets in 3T3-L1 preadipocytes^18,41^ and human liver cell culture.^16^ Similar to the other cell lines, EHDPP significantly increased lipid droplet formation in the adrenal cells in vitro. Quantification of the intracellular lipid profile of H295R cells upon exposure to EHDPP revealed dysregulation of several lipid species, including glycerolipids, glycerophospholipids, sphingolipids, and sterol esters similar to our previous observation in hepatospheroids cell culture.^17^ Exposure to EHDPP significantly increased the TG and LPC, LPC O, and decreased intracellular levels of HexCer, Hex2Cer, CER, and SM species. In contrast, a recent study reports OPFRs house dust mixture-mediated increase in the CER and HexCer levels in the adrenal cells (H295R),^42^ which could be related to synergistic or antagonistic interactions of OPFRs (or other components) of the complex studied mixtures. EHDPP as a single compound could have a higher potency in disrupting lipid homeostasis, leading to a more significant reduction in CER and HexCer levels in comparison to the complex mixture, where it formed only a minor fraction (1.18%) of the overall sample.^42^ Sphingolipids are essential components of cellular membranes and act as signaling molecules. Sphingolipids such as CER, sphingosine-1-phosphate (S1P), and SM have demonstrated their ability to modulate the steroid biosynthetic pathway at various stages.^43^ This modulation includes the regulation of steroidogenic gene expression and activity and their role as second messengers in signaling cascades.^43,44^ A noteworthy observation suggests that exposure to EHDPP leads to an elevation in saturated and monounsaturated lipids and a reduction in lipids containing unsaturated acyl chains. This phenomenon may be attributed to the potential inhibition of mRNA expression of SCD1 that plays a pivotal role in the synthesis of unsaturated lipids.^45^ Along with the sphingolipid metabolic pathway, which has a well-defined role in adrenal steroidogenesis, other lipids, such as phospholipids and sterols, were also altered after EHDPP exposure. The principal membrane phospholipid species, including PC, PE, and PS, were affected, and lysophospholipids significantly increased. These observations imply that EHDPP might affect the lipid rafts, which are composed of sphingolipids and cholesterol in the outer exoplasmic leaflet, connected to phospholipids and cholesterol in the inner cytoplasmic leaflet of the lipid bilayer.^46^ Phospholipids, including PI and PC, are known to influence adrenal cell signaling and, consequently, aldosterone biosynthesis, which links observed modulations of enzymes, including CYP11B2, critical in aldosterone production^47,48^ Interestingly, CE, the major constituent of the adrenal glands that serve as a pool for FC, which acts as a precursor for synthesizing all steroid hormones, was significantly downregulated. At the same time, no significant change in FC and TC (FC + CE) was observed. This observation indicates EHDPP-mediated defects in lipid storage, particularly in cholesterol metabolism. To decipher the mechanisms for defective cholesterol esterification, we measured the expression of ACAT, which was significantly down-regulated, indicating inefficient conversion of FC to CE after EHDPP exposure. The decrease in the total CE also indicates enhanced cholesterol esterase activity contributing to the total cholesterol pool, which should be further explored. Neutral lipids, such as triglycerides and cholesterols, are stored in most tissues as lipid droplets. Our staining (BODIPY 493/503) also showed an enhanced accumulation of neutral lipids with increasing concentrations of EHDPP. The acculturation of neutral lipid droplets was also demonstrated earlier in mouse preadipocytes^18,41^ and human liver cell culture.^16^ Simultaneously, the alterations in lipid metabolism imply a broader impact of EHDPP on lipid homeostasis within the cells. The intricate connection between hormone levels and lipid metabolism is well-established, as lipids play a crucial role in hormone synthesis and function. Hormonal regulation often involves feedback loops, and changes in lipid metabolism can impact the availability of substrates for hormone synthesis.^49^ Since steroid hormones are mainly synthesized in the adrenal gland, gonads, and placenta under the control of the hypothalamus–pituitary–adrenal-gonads, and placenta axis, respectively,^50^ future studies should aim to delve deeper into the intricate interactions within this hormonal axis using relevant animal models such as zebrafish or rodents. Moreover, given the observed alterations in lipid metabolism alongside changes in steroid hormone levels, future research should explore the crosstalk between lipid and hormone pathways.

## Supporting Information Available

Additional information regarding lipid internal standards and primer sequence (PDF)
