# Supplementary material for: 2-Ethylhexyl Diphenyl Phosphate Affects Steroidogenesis and Lipidome Profile in Human Adrenal (H295R) Cells
Source: Chem Res Toxicol. 2025 Apr 3;38(4):733–44. doi: 10.1021/acs.chemrestox.5c00030 (PMC12015954; doi:10.1021/acs.chemrestox.5c00030)
Supplement: Supplementary file 1 — tx5c00030_si_001.pdf [file tx5c00030_si_001.pdf]

Supplementary information

2-Ethylhexyl Diphenyl Phosphate affects steroidogenesis and lipidome profile in human adrenal (H295R) cells

Chander K. Negi[1], Darshak Gadara[1], Lola Bajard[1], Zdenek Spacil, and Ludek Blaha*[1]

1 RECETOX, Faculty of Science, Masaryk University, Kotlarska 2, 61137 Brno, Czech Republic

Address for correspondence:

*Prof. Ludek Blaha, Ph.D.

Email: ludek.blaha@recetox.muni.cz

RECETOX, Faculty of Science, Masaryk University,

Kotlarska 2, 61137 Brno, Czech Republic.

Number of pages: 11

Number of tables: 5

Table S1: Primer pairs used in real-time quantitative polymerase chain reactions.

Gene	Forward primer	Reverse primer
18S rRNA	CGTCTGCCCTATCAACTTTCG	TGCCTTCCTTGGATGTGGTAG
β-actin	CACTCTTCCAGCCTTCCTTCC	AGGTCTTTGCGGATGTCCAC
CYP11A	GAGATGGCACGCAACCTGAAG	CTTAGTGTCTCCTTGATGCTGGC
CYP11B2	TCCAGGTGTGTTCAGTAGTTCC	GAAGCCATCTCTGAGGTCTGTG
CYP17	AGCCGCACACCAACTATCAG	TCACCGATGCTGGAGTCAAC
CYP19	AGGTGCTATTGGTCATCTGCTC	TGGTGGAATCGGGTCTTTATGG
CYP21	CGTGGTGCTGACCCGACTG	GGCTGCATCTTGAGGATGACAC
3βHSD2	TGCCAGTCTTCATCTACACCAG	TTCCAGAGGCTCTTCTTCGTG
17βHSD1	CTCCCTCTGACCAGCAACC	TGTGTCTCCCACGCAATCTC
17βHSD4	TGCGGGATCACGGATGACTC	GCCACCATTCTCCTCACAACTC
StAR	GTCCCACCCTGCCTCTGAAG	CATACTCTAAACACGAACCCCACC
HMGR	TGCTTGCCGAGCCTAATGAAAG	AGAGCGTTCGTGGGTCCAT
ACAT2	CTTTAGCACGGATAGTTTCCTGG	GCTGCAAAGGCTTCATTGATTTC
ACAT1	ATGCCAGTACACTGAATGATGG	GATGCAGCATATACAGGAGCAA
SCD1	CCTTATGACAAGAACATTAGCCCC	GGTGAAGTTGATGTGCCAGC

Table S2: List of lipid internal standards used for lipidomic analysis using LC-MS/MS.

	**Lipid Standards**	**Final con. (ng/mL)**
1	15:0-18:1(d7) PC	416
2	15:0-18:1(d7) PE	13
3	15:0-18:1(d7) PG	78
4	17:0-14:1 PS	50
5	15:0-18:1(d7) PI	26
6	18:1(d7) Lyso PC	65
7	18:1(d7) Lyso PE	13
8	18:1(d7) Chol Ester	910
9	15:0-18:1(d7) DAG	26
10	15:0-18:1(d7)-15:0 TAG	143
11	d18:1-18:1(d9) SM	78
12	C16 (d3) Carnitine	0.25
13	d18:1/12:0 Cer	25
14	d18:1/12:0 Lac Cer	25
15	d18:1/12:0 Gla Cer	25
16	Cholesterol (d7)	12500

Table S3: Lipid classification as per LIPID MAPS consortium

**Lipid categories**	**Lipids subclasses**
Fatty Acyls	Acylcarnitine (CAR)
Sterol lipids	Cholesteryl esters (CE)
	Cholesterol (FC)
Glycerolipids	Diacylglycerols (DG)
	Triacylglycerols (TG)
Glycerophospholipids	Phosphatidylcholine (PC)
	Phosphatidylethanolamines (PE)
	Alkenylphosphatidylcholine (PC P)
	Alkylphosphatidylcholine (PC O)
	Lysophosphatidylethanolamines (LPE)
	Lysophosphatidylcholine (LPC)
	Phosphatidylglycerol (PG)
	Phosphatidylinositol (PI)
	Phosphatidylserine (PS)
Sphingolipids	Dihydroceramides (dhCer)
	Ceramides (CER)
	Hexosylceramide (HexCer), Hex2Cer
	Sphingomyelins (SM)

Table S4: Characterized lipid classes and species

	Lipid Classes	Number of Species
1	CAR	5
2	CE	11
3	Cholesterol	1
4	CER	17
5	DG	12
6	dhCer	8
7	Hex2Cer	3
8	HexCer	7
9	LPC	7
10	LPC-O	3
11	LPE	1
12	PC	36
13	PC-O	20
14	PC-P	8
15	PE	18
16	PE-O	6
17	PG	3
18	PI	14
19	PS	11
20	SM	23
21	TG	47

Table S5: SRM transitions for lipid species with respective precursor ion (Q1), product ion (Q3) and collision energy

**Lipid Species**	**Precursor Ion (Q1)**	**Product Ion (Q3)**	**Collision Energy (eV)**
CAR(20:0)	456,4	85,1	25
CAR(16:0)	400,4	85,1	25
CAR(16:1)	398,3	85,1	25
CAR(18:0)	428,4	85,1	25
CAR(18:1)	426,4	85,1	25
CE(18:1)	668,6	369,3	5
CE(18:2)	666,6	369,3	5
CE(18:3)	664.6	369.3	5
CE(20:1)	696,7	369,3	5
CE(20:2)	694,7	369,3	5
CE(20:3)	692,6	369,3	5
CE(20:4)	690,6	369,3	5
CE(20:5)	688.6	369.3	5
CE(22:1)	724,7	369,3	5
CE(22:4)	718.7	369.3	5
CE(22:6)	714,6	369,3	5
Cholesterol	369,4	81,0	45
Cholesterol	369,4	147,0	29
Cer(d16:1/16:0)	510.6	236.3	33
Cer(d16:1/18:0)	538.6	236.3	33
Cer(d16:1/22:0)	594,6	236,3	33
Cer(d16:1/24:1)	620,6	236,3	33
Cer(d18:1/14:0)	510,5	264,3	33
Cer(d18:1/16:0)	538,5	264,3	33
Cer(d18:1/18:0)	566,6	264,3	33
Cer(d18:1/20:0)	594,6	264,3	33
Cer(d18:1/22:0)	622,6	264,3	33
Cer(d18:1/23:0)	636,6	264,3	33
Cer(d18:1/24:0)	650,6	264,3	33
Cer(d18:1/24:1)	648,6	264,3	33
Cer(d18:2/16:0)	536.5	262.3	33
Cer(d18:2/18:0)	564.6	262.3	33
Cer(d18:2/20:0)	592.6	262.3	33
Cer(d18:2/22:0)	620.6	262.3	33
Cer(d18:2/24:1)	646.6	262.3	33
DG(14:0/16:0)	558,5	313,3	21
DG(16:0/16:1)	584,5	313,2	21
DG(16:0/18:1)	612,6	313,3	21
DG(16:0/18:2)	610,5	313,2	21
DG(16:1/18:1)	610,5	339,2	21
DG(18:0/18:1)	640,6	341,3	21
DG(18:0/18:2)	638,6	341,3	21
DG(18:0/20:4)	662,6	341,3	21
DG(18:1/18:1)	638,6	339,3	21

DG(18:1/18:2)	636,6	339,3	21
DG(18:1/20:4)	660,6	339,3	21
DG(18:1/20:3)	662.6	339.3	21
dhCer(d18:0/16:0)	540,5	284,3	33
dhCer(d18:0/18:0)	568,6	284,3	33
dhCer(d18:1/24:1)	648,6	282,3	33
dhCer(d18:1/18:0)	566.6	282.3	33
dhCer(d18:1/20:0)	594.6	282.3	33
dhCer(d18:1/22:0)	622.6	282.3	33
dhCer(d18:1/24:0)	650.7	282.3	33
dhCer(d18:1/24:1)	648.6	282.3	33
Hex2Cer(d18:1/16:0)	862,6	264,3	41
Hex2Cer(d18:1/22:0)	946.7	264.3	41
Hex2Cer(d18:1/24:0)	974,8	264,3	41
HexCer(d18:1/16:0)	700,6	264,3	41
HexCer(d18:1/18:0)	728,6	264,3	41
HexCer(d18:1/20:0)	756,6	264,3	41
HexCer(d18:1/22:0)	784,7	264,3	41
HexCer(d18:1/24:0)	812,7	264,3	41
HexCer(d18:1/24:1)	810,7	264,3	41
LPC(14:0)	468,3	184,1	29
LPC(16:0)	496,3	184,1	29
LPC(16:1)	494,3	184,1	29
LPC(18:0)	524,4	184,1	29
LPC(18:1)	522,4	184,1	29
LPC(18:2)	520,3	184,1	29
LPC(20:1)	550,4	184,1	29
LPC O-16:0	482.4	184.1	29
LPC O-18:0	510.4	184.1	29
LPC O-18:1	508.4	184.1	29
LPE(18:0)	482,3	341,3	25
PC(28:0)	678,5	184,1	37
PC(30:0)	706,5	184,1	37
PC(30:1)	704,5	184,1	37
PC(30:2)	702,5	184,1	37
PC(31:0)	720,5	184,1	37
PC(31:1)	718,5	184,1	37
PC(32:0)	734,6	184,1	37
PC(32:1)	732,5	184,1	37
PC(32:2)	730,5	184,1	37
PC(33:1)	746,6	184,1	37
PC(33:2)	744,6	184,1	37
PC(34:0)	762,6	184,1	37
PC(34:1)	760,6	184,1	37
PC(34:2)	758,6	184,1	37
PC(34:3)	756.5	184.1	37
PC(34:4)	754,5	184,1	37
PC(35:1)	774,6	184,1	37
PC(35:2)	772,6	184,1	37

PC(36:1)	788,6	184,1	37
PC(36:2)	786,6	184,1	37
PC(36:4)	782,6	184,1	37
PC(36:5)	780,5	184,1	37
PC(38:2)	814,6	184,1	37
PC(38:3)	812,6	184,1	37
PC(38:4)	810,6	184,1	37
PC(38:5)	808,6	184,1	37
PC(38:6)	806,6	184,1	37
PC(38:7)	804,5	184,1	37
PC(40:1)	844,7	184,1	37
PC(40:2)	842,7	184,1	37
PC(40:3)	840.6	184.1	37
PC(40:4)	838.6	184.1	37
PC(40:5)	836.6	184.1	37
PC(40:7)	832,6	184,1	37
PC(O-28:0)	664.5	184.1	37
PC(O-30:0)	692,6	184,1	37
PC(O-30:1)	690,5	184,1	37
PC(O-32:0)	720.6	184.1	37
PC(O-32:1)	718.6	184.1	37
PC(O-32:2)	716.6	184.1	37
PC(O-33:2)	736,6	184,1	37
PC(O-34:0)	748.6	184.1	37
PC(O-34:1)	746,6	184,1	37
PC(O-34:2)	744,6	184,1	37
PC(O-36:1)	774,6	184,1	37
PC(O-36:2)	772,6	184,1	37
PC(O-36:4)	768,6	184,1	37
PC(O-38:1)	802.7	184.1	37
PC(O-38:2)	800.6	184.1	37
PC(O-38:3)	798.6	184.1	37
PC(O-38:4)	796.6	184.1	37
PC(O-38:5)	794,6	184,1	37
PC(O-38:6)	792.6	184.1	37
PC(P-32:0)	718.5	184.1	37
PC(P-34:0)	746.6	184.1	37
PC(P-34:1)	744.6	184.1	37
PC(P-34:2)	742.5	184.1	37
PC(P-36:2)	770.6	184.1	37
PC(P-36:4)	766,5	184,1	37
PC(P-36:3)	768,5	184,1	37
PC(P-38:5)	792,6	184,1	37
PE(32:0)	692.5	551.5	25
PE(32:1)	690,5	549,5	25
PE(34:1)	718,5	577,5	25
PE(34:2)	716,5	575,5	25
PE(34:3)	714.5	573.5	25
PE(35:1)	732.5	591.5	25

PE(35:2)	730,5	589,5	25
PE(36:1)	746,6	605,6	25
PE(36:2)	744,5	603,5	25
PE(36:4)	740,5	599,5	25
PE(36:5)	738.5	597.5	25
PE(38:3)	770,6	629,6	25
PE(38:4)	768,5	627,5	25
PE(38:6)	764,5	623,5	25
PE(40:4)	796,6	655,6	25
PE(40:3)	798.6	657.6	25
PE(40:5)	794,6	653,6	25
PE(40:7)	790.5	649.5	25
PE (O-34:2)	702.5	561.5	25
PE (O-36:2)	730.6	589.6	25
PE (O-36:3)	728.5	587.5	25
PE (O-36:5)	724.5	583.5	25
PE (O-36:6)	722.5	581.5	25
PE (O-38:3)	756.6	615.6	25
PI(32:0)	828.5	551.5	17
PI(32:1)	826,5	549,5	17
PI(34:1)	854,5	577,5	17
PI(34:2)	852,5	575,5	17
PI(36:1)	882,6	605,6	17
PI(36:2)	880,5	603,5	17
PI(36:3)	878,5	601,5	17
PI(36:4)	876,5	599,5	17
PI(38:3)	906,6	629,6	17
PI(38:4)	904,5	627,5	17
PI(38:5)	902,5	625,5	17
PI(40:4)	932,6	655,6	17
PI(40:5)	930,6	653,6	17
PI(40:6)	928,5	651,5	17
PS(34:1)	854.5	577.5	17
PS(34:2)	760,5	575,5	25
PS(36:0)	792.6	607.6	25
PS(36:1)	790.5	605.5	25
PS(36:2)	788,5	603,5	25
PS(38:1)	818.6	633.6	25
PS(38:2)	816.6	631.6	25
PS(38:3)	814,5	629,5	25
PS(38:4)	812,5	627,5	25
PS(40:4)	840.6	655.6	25
PS(40:5)	838.5	653.5	25
PG(32:1)	738.5	549.5	21
PG(34:1)	766.5	577.5	21
PG(34:2)	764,5	575,5	21
PG(36:2)	792,5	603,5	21
SM(31:1)	661,5	184,0	25
SM(32:0)	677,6	184,0	25

SM(32:1)	675,5	184,0	25
SM(32:2)	673,5	184,0	25
SM(33:1)	689,6	184,0	25
SM(34:0)	705,6	184,0	25
SM(34:1)	703,6	184,0	25
SM(34:2)	701,6	184,0	25
SM(35:1)	717.6	184	25
SM(36:1)	731,6	184,0	25
SM(37:1)	745.6	184	25
SM(38:1)	759,6	184,0	25
SM(38:3)	755,6	184,0	25
SM(40:0)	789,7	184,0	25
SM(40:1)	787,7	184,0	25
SM(40:2)	785,7	184,0	25
SM(40:3)	783,6	184,0	25
SM(41:0)	803,7	184,0	25
SM(41:1)	801,7	184,0	25
SM(41:2)	799,7	184,0	25
SM(42:1)	815,7	184,0	25
SM(42:2)	813,7	184,0	25
SM(43:2)	827,7	184,0	25
SM(44:2)	841,6	184,0	25
TG 42:0/NL-14:0	740.7	495.5	21
TG 44:0/NL-14:0	768.7	523.5	21
TG 44:1/NL-14:0	766.7	521.5	21
TG 46:0/NL-14:0	796,7	551,5	21
TG 46:1/NL-14:0	794,7	549,5	21
TG 46:2/NL-14:0	792,7	547,5	21
TG 48:0/NL-16:0	824,8	551,5	21
TG 48:1/NL-18:1	822,8	523,5	21
TG 48:2/NL-18:1	820,8	521,5	21
TG 48:2/NL-14:1	820.8	577.6	21
TG 48:2/NL-16:0	820.8	547.5	21
TG 48:2/NL-16:1	820.8	549.5	21
TG 48:2/NL-18:1	820.8	521.5	21
TG 48:3/NL-16:1	818,8	547,5	21
TG 48:3/NL-18:2	818.8	521.5	21
TG 49:1/NL-16:0	836,8	563,5	21
TG 49:1/NL-18:1	836.8	537.5	21
TG 50:0/NL-18:0	852,8	551,5	21
TG 50:1/NL-14:0	850,8	605,6	21
TG 50:1/NL-18:1	850,8	551,5	21
TG 50:2/NL-16:0	848,8	575.5	21
TG 50:2/NL-18:1	848,8	549,5	21
TG 50:2/NL-18:2	848,8	551,5	21
TG 50:3/NL-16:1	846,8	575,6	21
TG 50:3/NL-14:1	846.8	603.6	21
TG 50:3/NL-16:1	846.8	575.6	21
TG 50:3/NL-18:1	846,8	547,5	21

TG 51:1/NL-18:1	864,8	565,5	21
TG 51:2/NL-15:0	862,8	603,6	21
TG 51:2/NL-16:0	862.8	589.6	21
TG 51:2/NL-18:1	862,8	563,5	21
TG 52:0/NL-16:0	880,8	607,5	21
TG 52:1/NL-18:0	878,8	577,5	21
TG 52:2/NL-16:0	876,8	603,6	21
TG 52:3/NL-16:1	874,8	603,6	21
TG 52:3/NL-18:2	874.8	577.6	21
TG 52:4/NL-16:0	872.8	599.6	21
TG 52:4/NL-18:1	872,8	573,6	21
TG 53:2/NL-17:0	890,8	603,6	21
TG 54:2/NL-18:0	908,9	607,6	21
TG 54:3/NL-18:1	906,9	607,6	21
TG 54:4/NL-18:0	904,9	603,6	21
TG 54:3/NL-18:1	902,9	603,6	21
TG 54:4/NL-18:0	900.8	599.5	21
TG 54:4/NL-18:2	900,9	603,9	21
TG 54:5/NL-18:1	898.9	599.6	21
TG 54:6/NL-18:2	896.9	599.6	21
TG 58:3/NL-22:1	958,8	603,5	21
